# Spinal 5-HT_3_ receptor contributes to somatic hyperalgesia induced by sub-chronic stress

**DOI:** 10.1177/1744806919859723

**Published:** 2019-07-05

**Authors:** Zhuo-Lin Li, Yang Xue, Zhuo-Ying Tao, Wen-Zhi Du, Yue-Gui Jiang, Dong-Yuan Cao

**Affiliations:** 1 Key Laboratory of Shaanxi Province for Craniofacial Precision Medicine Research, Research Center of Stomatology, Xi’an Jiaotong University College of Stomatology, Xi’an, Shaanxi, P. R. China; 2 Department of Endodontics, Xi’an Jiaotong University College of Stomatology, Xi’an, Shaanxi, P. R. China

**Keywords:** Pain, stress-induced hyperalgesia, forced swim, descending facilitation, 5-HT_3_ receptor, spinal cord

## Abstract

Stress facilitates pain perception and sensitizes pain pathways, but the underlying mechanism is still unclear. The purpose of this study was to investigate whether the activation of 5-hydroxytryptamine (5-HT) subtype-3 receptor in the spinal cord contributes to somatic hyperalgesia induced by repeated three-day forced swim in the estradiol replacement rats after ovariectomy. Somatic sensitivity was assessed by thermal withdrawal latency to radiant heat and mechanical withdrawal threshold to von Frey filaments. The expression of 5-HT_3A_ receptor in the L4–L5 dorsal spinal cord was examined by Western blot. Repeated forced swim stress reduced the thermal withdrawal latency and mechanical withdrawal threshold, and the presence of estradiol exaggerated this hyperalgesia. The expression of 5-HT_3A_ receptor in the L4–L5 dorsal spinal cord increased significantly following repeated forced swim in estradiol replacement rats. Intrathecal injection of 5-HT_3_ receptor antagonist Y-25130 blocked the somatic hyperalgesia induced by forced swim stress. These data indicate that 5-HT_3_ receptor activation through the descending facilitation system contributes to the somatic hyperalgesia evoked by forced swim stress. The results may provide a new therapeutic avenue for alleviating pain induced by stress.

## Introduction

Stress affects brain activities and promotes long-term changes in the nervous system. Previous studies have shown that exposure to an acute, robust, and intense stress leads to a reduction in pain responses, a phenomenon described as stress-induced analgesia.^1^ On the other hand, repeated or chronic exposure to physical or psychological stressors often causes stress-induced hyperalgesia in humans and experimental animals.^2–4^ Unfortunately, the mechanism accounting for the stress-induced hyperalgesia is unclear; therefore, there is no efficacious treatment for the related pain.

The forced swim (FS) test is usually used to assess depression-like behaviors to examine “hopelessness” of rats and mice. The FS stress is thought to be a psychophysical stress since the animals need to cope with fear of drowning psychologically and to avoid water suffocation physically.^5–7^ It has been shown that repeated FS stress for three days evokes a delayed and long-lasting (eight to nine days) thermal and chemical cutaneous hyperalgesia and an increase of c-Fos expression in the spinal dorsal horn,^6,8^ as well a significant increase in the pain scores during formalin test in both the early and late phases in male rats.^5^ An electrophysiological study showed that repeated FS stress increased medullary dorsal horn neuron activities to noxious temporomandibular joint stimuli in female rats.^9^ Our previous studies showed that repeated FS stress induced visceral hypersensitivity in intact female rats.^10^ These data suggest that repeated FS for three days is a reliable sub-chronic stress model which mimics the human chronic pain condition and provides a platform for us to further understand the behavioral and neurochemical basis of stress-induced hyperalgesia.

Recent studies indicate that behavioral hypersensitivity and neuronal hyperexcitability in the central nervous system (CNS) in animal models of persistent pain are closely linked to long-lasting activation of the descending modulatory circuits.^11,12^ Serotonin (5-hydroxytryptamine, 5-HT) from the descending pain modulatory pathway is critical to nociceptive processing.^12^ It is well established that the descending serotonergic system from the rostral ventromedial medulla (RVM) in the brainstem is involved in the modulation of spinal nociceptive transmission.^13^ There is evidence for both pronociceptive and antinociceptive effects of 5-HT in behavioral and electrophysiological paradigms.^14–16^ The effects of 5-HT on nociception depend on the subtype of receptors activated by this amine and the localization (central or peripheral) of the receptors in the nervous system.^17^

The 5-HT receptors belong to a family of seven receptors (5-HT_1-7_) which are subdivided into 14 subtypes.^18^ In contrast to the well-established inhibitory role of this system in pain processing, some consistent experimental data have indicated a nociceptive role for serotonin and its contribution to the pain descending facilitatory pathway, through the activation of 5-HT_3_ receptor, mainly in the spinal cord.^11,12,16^

The 5-HT_3_ receptor is a cation-selective ion channel of the Cys-loop superfamily.^19^ Two subunits of the receptor exist which have been termed as 5-HT_3A_ and 5-HT_3B_.^19^ All functional 5-HT_3_ receptors are homomers of 5-HT_3A_ subunits or heteromers of 5-HT_3A_ and 5-HT_3B_ subunits. In the peripheral nervous system, heteromeric receptors are likely to be common, whereas receptors in the CNS appear to be composed of only 5-HT_3A_ subunits.^20^

It has been demonstrated that spinal 5-HT_3_ receptor contributes to the descending pain facilitation in the development of inflammatory pain and neuropathic pain.^12,15,21^ However, it is unknown whether the descending pain facilitation is involved in the development of somatic hyperalgesia induced by sub-chronic stress. The aim of this study was to examine whether 5-HT_3_ receptor in the spinal cord plays an important role in somatic hyperalgesia induced by three-day FS stress.

## Materials and methods

### Animals

Experimental protocols were approved by the Institutional Animal Care and Use Committees of Xi’an Jiaotong University, China, and adhered to guidelines for experimental pain in animals published by the International Association for the Study of Pain. Female Sprague-Dawley (SD) rats weighing 200–230 g were obtained from Xi’an Jiaotong University Laboratory Animal Center (Xi’an, Shaanxi, China). Rats were housed in pairs with free access to food and water with 12 h/12 h alternating light–dark cycle.

### Surgery

**Figure 1.**
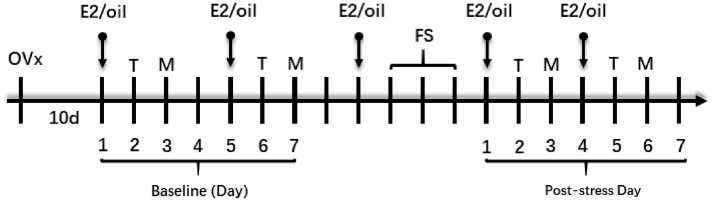
Experimental design. All rats were OVx. After 10 days of recovery, E2 or oil was injected subcutaneously every four days. The baselines of thermal withdrawal latency (T) and mechanical withdrawal threshold (M) were tested before FS. From day 2 post last FS, thermal withdrawal latency and mechanical threshold were measured on the second and third days, respectively, after each E2/oil injection. The measurements were continued until the thermal withdrawal latency or mechanical threshold returning to the baseline level. OVx: ovariectomized; E2: estradiol; FS: forced swim.

The experimental procedures are shown in Figure 1. Rats were ovariectomized (OVx) by a dorsolateral approach with inhalation anesthesia by 2%–3% isoflurane in a gas mixture of O_2_.^22^ Ten days after OVx, rats were injected subcutaneously with 17-β-estradiol (E2, 50 µg in 100 µL safflower oil, Sigma, St Louis, MO, USA) or safflower oil (vehicle, 100 µL). The E2 injection was repeated at four-day intervals to mimic the fluctuation of a normal estrous cycle.^22^

### FS stress

Rats were forced to swim in the second day post E2 injection after baseline tests for thermal withdrawal latency and mechanical withdrawal threshold. In detail, rats were placed in a cylindrical container (30 cm diameter) filled to 20 cm with 25°C–26°C water. Rats swam for 10 min on the first day and 20 min on the following two days.^23^ After each session, rats were dried in a heated area before being returned to their home cages. FS was carried out at the same time in the morning, 9:00 a.m. to noon, to avoid any influence of circadian rhythms. The rats in the control group for FS remained undisturbed in their home cages. Therefore, a little bit of additional stress may be produced from handling and drying for the FS rats compared to the rats in the control group. The day after the last FS was designated day 1 post FS. The behavioral examiners were blinded.

### Thermal withdrawal latency and mechanical withdrawal threshold

The thermal withdrawal latency and mechanical withdrawal threshold were performed on the second and third days, respectively, after each E2 injection (Figure 1). The right hindpaw was tested for the thermal withdrawal latency and left for mechanical withdrawal threshold to avoid the interference of thermal test in mechanical test. The baseline values were measured twice to ensure the reproducibility, and the average of the two baseline values was used for analysis.

Thermal pain was assessed by measuring the latency of the right hindpaw withdrawal in response to a radiant heat source.^24^ Animals were placed in clear plexiglass chambers on an elevated table and allowed to acclimate for 30 min. An infrared (IR) beam was applied from underneath the glass floor and focused on the plantar surface of right hindpaw, and the paw withdrawal latency was recorded. The IR intensity was adjusted to produce mean baseline paw withdrawal latencies ranging between 10 s and 12 s. A 20-s cutoff was used to avoid excessive tissue injury. Three records were collected per animal with an inter-stimulus interval of 5 min. The average of the three trials was then determined.

The mechanical sensitivity was measured with a series of calibrated von Frey filaments.^25^ Rats were placed in individual plexiglass chambers with wire mesh floors and transparent covers (20 × 20 × 25 cm). Behavioral accommodation was allowed for approximately 30 min, until cage exploration and major grooming activities ceased. The mechanical withdrawal threshold was determined using the up-down paradigm. The test for each rat started with a specific von Frey filament to manually press perpendicularly against the left hindpaw plantar surface. In the absence of a paw withdrawal response to the initially selected filament, a stronger stimulus was then selected. If there is a positive response, the next weaker stimulus was picked. Counting of the critical six data points did not begin until the response threshold was first crossed, at which time the two responses straddling the threshold were retrospectively designated as the first two responses of the series of 6.

### Intrathecal injection

A lumbar puncture procedure was adapted according to the previous study.^12^ Briefly, rats for intrathecal injection were anesthetized with isoflurane as mentioned above. After shaving the tail part of the rats’ back, they were placed in a prone position with a round tube underneath the abdomen. A disposable 25-gauge 1-in. needle connected to a 25 µL Luer tip Hamilton syringe was inserted slowly at the intervertebral space between the L4 and L5 vertebrae and the needle was allowed to penetrate the dura. A quick flick of the tail indicated the needle entering into the intrathecal space. Then 5-HT_3_ receptor antagonist Y-25130 hydrochloride (Tocrics Bioscience, Bristol, UK) was injected slowly in 2 min (30 fmol/10 µL). The previous study showed that intrathecal injection of 30 fmol of Y-25130 alone did not affect the baseline of thermal and mechanical sensitivity in SD rats.^12^ Intrathecal injection was performed 30 min before each FS and before E2 injection at one day prior to and post FS.

### Western blot

The second day following the last FS, rats for Western blot experiments were anesthetized with isoflurane (5%) and decapitated. The spinal cord was removed by pressure ejection with ice cold saline as previously described.^23^ The L4–L5 section of the spinal cord was isolated, and the dorsal half was separated and stored at −80°C until use.

Tissues were homogenized in radioimmunoprecipitation assay buffer (1% NP-40, 1% Sodium deoxycholate, 0.1% sodium dodecyl sulfate) and protease inhibitor cocktail (Boster, AR1182, Wuhan, China). The homogenates were centrifuged at 10,000 g for 10 min at 4°C and the supernatant was collected. Protein concentration in supernatants was measured by using the bicinchoninic acid method. After denaturing, protein samples were fractionated 18 µg per lane on 4%–12% sodium dodecyl sulfate-polyacrylamide gel electrophoresis gel and blotted to polyvinylidene difluoride membranes. The membranes were blocked in 5% nonfat milk for 2 h and then were incubated with primary antibody directed against 5-HT_3A_ receptor (1:300, Novus Biologicals, NB100-56351, Littleton, CO, USA) and against GAPDH (1:4000, Boster, BA2913) at 4°C overnight, respectively. The membranes were further incubated for 2 h in TBST buffer (10 mM Tris, 150 mM and 1 ml Tween-20 dissolved in 1 L distilled water with pH = 7.4–7.6) with goat anti-rabbit secondary antibody (Boster, BA1054) at 1:4000 dilution. The antigen–antibody complexes were visualized by enhanced chemiluminescence (Thermo Scientific, Waltham, MA, USA). The immunoreactive band densities were analyzed using Image J software.

### Data analysis

All data are presented as mean ± standard error of the mean. Statistical and figure analyses were performed using GraphPad Prism 6 software. One-way repeated measures analysis of variance (ANOVA) followed by Dunnett post hoc test was used for comparisons of means except Western blot data. One-way ANOVA followed by Dunnett post hoc test was used for protein expression of 5-HT_3A_ receptor. *P* < 0.05 was considered significant.

## Results

### Estrogen aggravates repeated FS-induced somatic hyperalgesia

**Figure 2.**
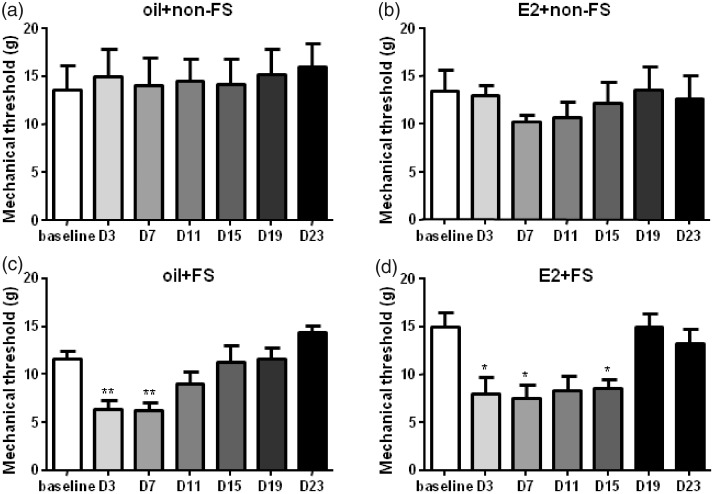
The mechanical thresholds decreased followed by FS stress combined with or without E2 injection compared to baseline. There were no significant differences in mechanical thresholds in the non-FS groups compared to baselines. *^, ^**P < 0.05, 0.01 versus baseline in each group, respectively. D3 indicates the third day post last FS. E2: estradiol; FS: forced swim.

**Figure 3.**
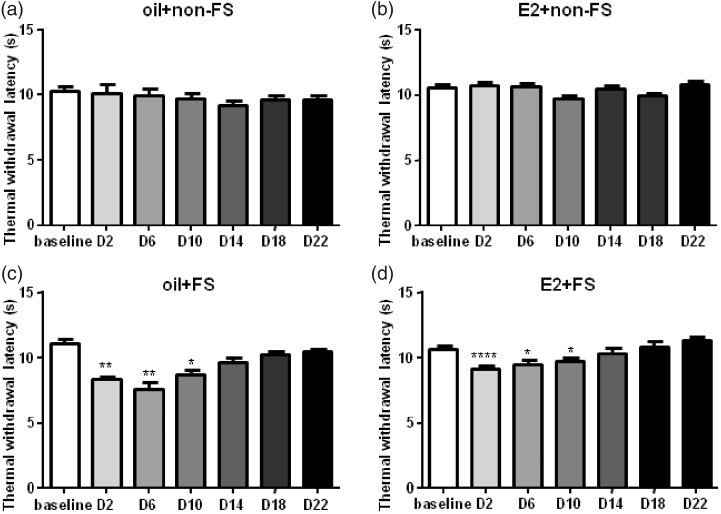
The thermal withdrawal latency decreased followed by FS stress combined with or without E2 injection compared to baseline. There were no significant differences in the thermal withdrawal latency in the non-FS groups compared to baseline. *^, ^**^, ^****P < 0.05, 0.01, 0.0001 versus baseline in each group, respectively. D2 indicates the second day post last FS. E2: estradiol; FS: forced swim.

The previous studies showed that three-day FS in male rats caused a delayed and long-lasting (eight to nine days) thermal and chemical cutaneous hyperalgesia.^6,7^ Three-day FS stress also evoked visceral hypersensitivity in female rats.^23,26^ Here, we found that repeated FS reduced thermal withdrawal latency and mechanical withdrawal threshold compared to baseline in E2 replacement female rats (Figures 2 and 3).

In the oil + FS group, mechanical hyperalgesia lasted for seven days (*F*_7,42_ = 8.019, *P* = 0.0023) after FS. The mechanical hyperalgesia lasted for 15 days after FS in the E2 replacement group (E2 + FS group, *F*_10,60_ = 7.756, *P* = 0.0007). We used safflower oil as vehicle control to clarify whether E2 strengthens stress’ effect on pain. The duration of mechanical hyperalgesia was longer in the E2 + FS group than that in the oil + FS group (15 vs. 7 days, Figure 2). Hargreaves test is widely used for assessing tolerance to thermal pain in rats. In our test, thermal hyperalgesia lasted for 10 days in both the oil + FS group (*F*_7,42_ = 13.40, *P* < 0.0001) and the E2 + FS group (*F*_10,60_ = 11.18, *P* < 0.0001, Figure 3). These data suggest that three-day repeated FS induces somatic hyperalgesia. No mechanical and thermal hyperalgesia occurred in the oil + non-FS (*F*_7,42_ = 0.8155, *P* = 0.5043 for mechanical withdrawal threshold and *F*_7,42_ = 1.681, *P* = 0.2003 for thermal withdrawal latency) and the E2 + non-FS groups (*F*_6,36_ = 0.5587, *P* = 0.6588 for mechanical threshold and *F*_6,36_ = 3.235, *P* = 0.0539 for thermal withdrawal latency), suggesting E2 alone does not induce mechanical and thermal hyperalgesia (Figures 2 and 3).

### Repeated FS stress in E2 replacement rats increases 5-HT_3A_ receptor expression in the spinal cord

**Figure 4.**
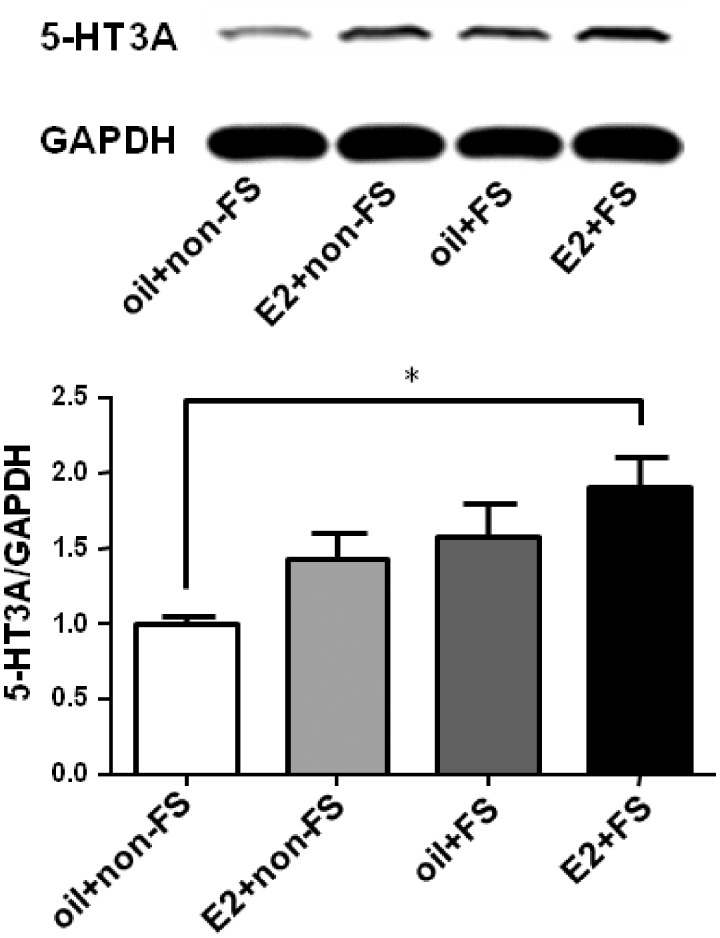
The expression of 5-HT_3A_ receptor increased in the E2 + FS group compared to that in the oil + non-FS group. *P < 0.05 versus the oil + non-FS group. E2: estradiol; FS: forced swim.

We have demonstrated that repeated FS stress induced somatic hyperalgesia and estrogen may exaggerate this hyperalgesia, but the mechanism is unclear. One possibility is that the hyperalgesia is caused by an activation and increase of pronociceptive proteins in the spinal cord. 5-HT_3_ receptor has been shown to be involved in the descending pain facilitation in many pain conditions. Therefore, we examined the protein expression of 5-HT_3A_ receptor in the L4–L5 dorsal spinal cord. The expression of 5-HT_3A_ receptor increased significantly in the E2 + FS group compared with that in the oil + non-FS group (*F*_3,16_ = 3.723, *P* = 0.0333, Figure 4), suggesting that 5-HT_3A_ receptor in the spinal cord and the descending pain facilitatory system are involved in the development of stress-induced hyperalgesia.

### Blocking 5-HT_3_ receptor in the spinal cord attenuates somatic hyperalgesia induced by repeated FS

**Figure 5.**
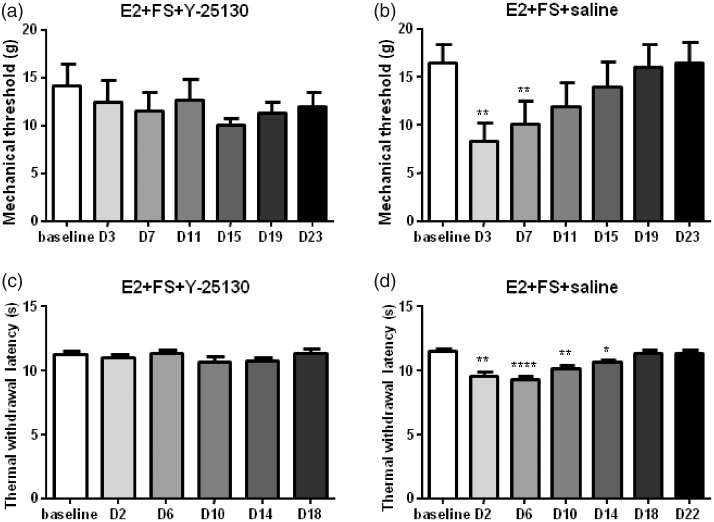
The thermal and mechanical hyperalgesia induced by repeated FS was blocked by intrathecal injection of 5-HT_3_ receptor antagonist Y-25130. *^, ^**^, ^****P < 0.05, 0.01, 0.0001 versus baseline in each group, respectively. E2: estradiol; FS: forced swim.

As Western blot data showed that the expression of 5-HT_3A_ receptor in the E2 + FS group significantly increased compared with that in the oil + non-FS group, we next tested whether 5-HT_3_ receptor antagonist could block the somatic hyperalgesia induced by FS stress in E2 replacement rats. When intrathecal injection of vehicle (saline) in the E2 + FS rats, repeated FS caused a mechanical threshold reduction for seven days after the FS (*F*_11,66_ = 9.676, *P* < 0.0001) and a reduction of thermal withdrawal latency for 14 days (*F*_11,66_ = 18.36, *P* < 0.0001). Intrathecal injection of 5-HT_3_ receptor antagonist Y-25130 for five consecutive days before each FS and injection of E2 blocked somatic hyperalgesia induced by FS in E2 replacement rats (*F*_7,42_ = 0.9743, *P* = 0.4178 for mechanical threshold and *F*_7,35_ = 1.397, *P* = 0.2720 for thermal withdrawal latency, Figure 5). The behavioral results combined with the Western blot data indicate that 5-HT_3_ receptor activation in the spinal cord contributes to somatic hyperalgesia induced by repeated FS stress in E2 replacement rats.

## Discussion

The FS stress is thought to be a psychophysical stress since the rats are psychologically panicked and physically exhausted.^5^ In this study, we used repeated FS as a stressor in female SD rats to evoke long-lasting mechanical and thermal hyperalgesia, which is consistent with previous studies. For example, repeated FS stress induced a significant reduction in escape behavior (learned helplessness).^8^ Three-day FS induced a delayed nine-day thermal hyperalgesia measured with the hot plate assay,^6,7,27,28^ chemical nociception measured with the formalin test,^5,8,29,30^ and musculoskeletal nociception measured using the grip force assay.^28^ The formalin-evoked chemical nociception behavior was almost completely prevented by the destruction of the RVM,^5^ indicating that the descending pain modulatory system is involved in stress-induced hyperalgesia. The FS stress significantly enhanced the complete Freund’s adjuvant-induced thermal hyperalgesia.^31^ In other experimental designs, five-day FS stress (5 min/day, 20 ± 1°C) in adult male Wistar rats produced hyperalgesia in tail-flick test and extremely elevated the plasma level of corticosterone, suggesting that repeated FS as an activator of the hypothalamic–pituitary–adrenal axis.^32^

FS increases the neuronal activity in the spinal cord as indicated by the increase in c-Fos in laminae I–VI of dorsal horn,^8,29,30^ decreases release of γ-aminobutyric acid in the spinal cord,^29,30^ the hippocampus and the thalamus–hypothalamus region,^33^ or increases NMDA activity in the spinal cord.^29^ FS also increases the expression of pCREB (phosphorylated cAMP-response element binding protein), DeltaFosB, and c-Fos in the locus coeruleus.^31,34,35^ The stress-induced hyperalgesia is prevented by the administration of relatively selective serotonin reuptake inhibitors (clomipramine and fluoxetine) and the serotonin precursor tryptophan, indicating that the central serotoninergic activity contributes to the hyperalgesia.^6,29^ However, to the best of our knowledge, there is no report on the changes in the expression of 5-HT receptors in the spinal cord after FS stress.

It is well known that the descending serotonergic system from the RVM in the brainstem is involved in the modulation of spinal nociceptive transmission. Neurons in the RVM are the major source of 5-HT in the dorsal horn. The descending serotonergic system arising in the RVM bidirectionally modulate dorsal horn activity through combining with different 5-HT receptors in the dorsal horn.^36^ Increasing evidence supports that spinal 5-HT_3_ receptors play a crucial role in cellular and molecular mechanisms in the development and maintenance of pain states.^12,36^

In this study, we found a significant increase in the expression of 5-HT_3A_ receptor in the L4–L5 spinal cord after FS. Selective blockage of 5-HT_3_ receptor in the spinal cord resulted in a reduction of nociceptive behavioral response evoked by repeated FS stress. The activation of serotonergic system is enhanced following formalin-induced inflammation and peripheral nerve injury, but not following carrageenan-induced inflammation.^37^ These results suggested that the activation of the descending facilitatory pathways may contribute to spinal excitability in some pain conditions. Thus, the RVM-spinal 5-HT system is implicated in the descending pain facilitation involving central mechanisms, and targeting this system may provide a novel avenue to manage pain.^38^

The previous studies showed that E2 exacerbated visceral pain in animals.^39^ Visceral hypersensitivity induced by stress persisted several weeks in females but only a few days in males, and facilitated by orchiectomy and injection of E2 in males.^26^ E2 replacement is an essential factor in increasing the expression of 5-HT_3A_ receptor in the spinal cord in this study, which is consistent with the observation that the development of somatic hyperalgesia in the E2 + FS group. However, repeated FS alone in this study also induced short period hyperalgesia in rats. Subcutaneous injection of E2 alone did not induce changes in the mechanical and thermal hyperalgesia in this study. This result was not in agreement with the previous studies showing that E2 exaggerated mechanical and thermal hyperalgesia. For example, An et al. found that intrathecal or intravenous injection of E2 caused rapid reduction in the mechanical pain threshold in OVx rats.^40^ Zhang et al. found that intrathecal injection of E2 produced mechanical allodynia and thermal hyperalgesia among male, female, and OVx rats.^41^ The discrepancy may be due to the different injection methods.

## Conclusion

In this study, three-day FS stress induced somatic hyperalgesia in E2 replacement rats and 5-HT_3_ receptor antagonist blocked the stress-induced hyperalgesia. The fact that stress exacerbates chronic pain provides novel therapeutics for pain management targeting the pain- and stress-responsive brain regions of the descending facilitatory system. Further studies are needed to elucidate whether specific brain nuclei such as RVM contribute to stress-induced hyperalgesia.
